# Visibility of video laryngoscope in high-illuminance environment simulating outdoor conditions: effects of screen conditions and polarized sunglasses

**DOI:** 10.1186/s40981-025-00778-9

**Published:** 2025-03-03

**Authors:** Takayuki Hasegawa, Rieko Oishi, Hidemi Ishido, Ayumi Oishi, Satoki Inoue

**Affiliations:** Department of Anesthesiology and Division of Intensive Care, Fukushima Medical University, 1 Hikarigaoka, Fukushima, 960-1295 Japan

**Keywords:** Video laryngoscope, High-illuminance environment, Visual analog scale

## Abstract

**Background:**

Video laryngoscopes generally increase the success rate of tracheal intubation and clinical outcome compared to traditional direct laryngoscopes. However, there is a concern that their effectiveness can be compromised in bright outdoor environments. The impact of polarized sunglasses on the visibility of a video laryngoscope in a high-illumination environment simulating outdoor conditions was assessed. Additionally, the effect of screen smudges on screen visibility was examined.

**Methods:**

A high-illumination environment was created using artificial light equivalent to daylight outdoors. Twenty-four anesthesiologists participated in this study. A commercially available laryngoscope was utilized to evaluate the visibility of the monitor screen and visualize the larynx. The experiment involved a fixed order sequence, including viewing with the naked eye, wearing sunglasses, cleaning the screen without wearing sunglasses, and cleaning the screen while wearing sunglasses, to evaluate visibility with each intervention. A visual analog scale (VAS) (0–100 mm) was used to evaluate the visibility of the larynx displayed on the screen.

**Results:**

Polarized sunglasses significantly enhanced visibility, with a median VAS score of 12 compared to 5 (*P* = 0.004). Moreover, cleaning the monitor screen significantly improved visibility more than wearing sunglasses alone, with a median VAS score of 38 compared to 12 (*P* = 0.002). Additionally, wearing sunglasses after cleaning the monitor screen provided even better visibility compared to only cleaning the screen, with a median VAS score of 57 compared to 38 (*P* = 0.002).

**Conclusions:**

Based on these findings, it is suggested that when using a video laryngoscope outdoors in sunny conditions, the first step to address impaired visibility should be to clean the screen. Wearing sunglasses, if possible, can also be effective in improving visibility.

## Introduction

Studies have shown that video laryngoscopes generally increase the success rate of tracheal intubation and clinical outcome compared to traditional direct laryngoscopes. [1, 2] However, there is a concern that their effectiveness can be compromised in bright outdoor environments. [3, 4] The use of video laryngoscopes in bright outdoor settings is primarily related to screen visibility. Factors such as bright sunlight can make it difficult to see the video screen clearly, which can impede the ability to perform tracheal intubation effectively. This is because the screens can suffer from the so-called glare, which is difficulty of seeing in the presence of bright light such as direct or reflected sunlight or artificial light, which make it hard for health providers to see the laryngeal structures clearly. Sunglasses are often worn in bright outdoor environments, which help to reduce glare. Of those, polarized sunglasses are designed to reduce glare and provide better clarity and contrast in bright outdoor conditions. Polarized sunglasses can improve screen visibility on the video laryngoscope. However, even minor screen smudges can enhance glare on the display, drastically reducing screen visibility in bright outdoor conditions.

In this study, a high-illumination environment equivalent to daylight outdoors was created using artificial light. The aim of this study was to evaluate the effect of polarized sunglasses on the visibility of the video laryngoscope in a high-illumination environment simulating outdoor conditions. Additionally, the study aimed to assess the impact of screen smudges on screen visibility under these high-illumination conditions.

## Methods

This study was registered with the University Hospital Medical Information Network Clinical Trials Registry (UMIN R000061898 registered on July 21, 2024). Given the nature of the study, the Fukushima Medical University Hospital Institutional Review Board waived the requirement for approval. However, verbal informed consent for participation was obtained from all the participants. Twenty-eight healthy volunteers, who had no visual disorders in previous biannual medical examinations, were recruited from consultant and resident anesthesiologists in this institute for the study. Individuals who use corrective eyewear including contact lens were included. However, ones who use sunglasses in their daily life were excluded.

### Experimental environment

The study was conducted in a high-illuminance environment simulating outdoor conditions to ensure consistent brightness for all participants. Maintaining such consistency is difficult in natural outdoor settings due to variations in time and weather. Given that daylight on a clear day typically ranges from around 50,000 to 100,000 lx, the goal was to replicate outdoor conditions using commercially available floodlights. Additionally, to simulate screen smudges, artificial sweat (1% emulsified saline solution) was used to recreate dirt on the screen.

The experiment was performed in a room of the hospital, which had no windows and was blocked from sunlight. The usual luminous intensity provided by the ceiling light ranged from 300 to 500 lx. To simulate outdoor conditions on a typical clear day with reproducibility, high-illuminance intensity (50,000–60,000 lx) was created on the monitor screen of the video laryngoscope (McGRATH MAC, Covidien Japan, Japan) using commercially available floodlights (B0C2T3JNV5, Aicdas, China). Briefly, with 5 floodlights, each capable of emitting a luminous flux of 950,000 lumens, attached to a stanchion using double clips, a high-illuminance environment without shadows on the monitor screen was created, and the illuminance intensity was adjusted to 50,000–60,000 lx using an illuminometer (VLT113, C-Timvasion, China). Finally, the set of floodlights was placed approximately 2.5 m away from the monitor screen of the laryngoscope. Regarding simulated screen smudges, the screen was swiped two or three times with a sheet of gauze soaked in artificial sweat and then dried using a hair dryer. While sweat primarily consists of NaCl, a 1% emulsified saline solution was used to replicate dirt on the screen from finger marks containing sebum.

### The object of evaluation for visibility

An adult patient upper body airway simulator (Ambu® Airway Management Trainer, Ambu A/S, Denmark), was used for evaluation for visibility. The laryngoscope blade (MacGrath disposable blade size 3) was fixed into position to visualize the larynx using a retort stand and a clamp [5] so that the same fixed laryngeal exposure. In this condition, screen smudges were made by the previously mentioned procedure. These smudges were designed to simulate those occurring during daily use, and it was confirmed that the screen visibility was not impaired by this level of smudging under normal luminous intensity.

### Experimental protocol

#### Confirmation of light adaptation

First, a warning was issued against directly viewing the floodlights during the examination. If a participant inadvertently looked at the floodlights, they were excluded from the study. To allow their eyes to acclimate to the brightness, participants were exposed to conditions of 50,000–60,000 lx for at least 1 min. To confirm light adaptation, participants were asked to read aloud large letters from a newspaper at a distance of 30–40 cm. If a participant was unable to read the letters, it was determined that they had not adapted to the high-illuminance environment, and they were excluded from the study.

#### ***Preparation for experimental settings (***Fig. 1***)***

**Fig. 1:**
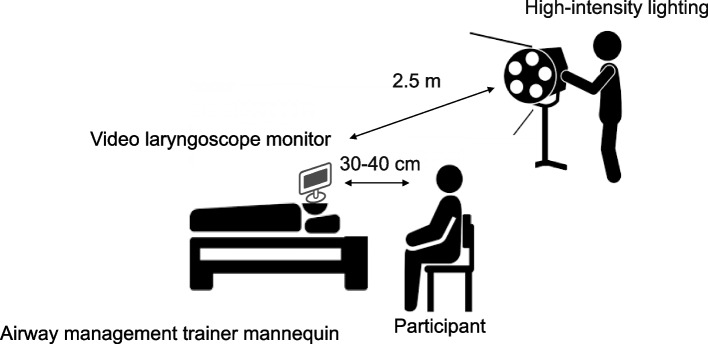
Experimental setting. The illuminance intensity was adjusted to 50,000–60,000 lx using five floodlights. The set of floodlights was placed approximately 2.5 m away from the monitor screen of the laryngoscope. The participants evaluated the screen visibility at a distance of 30–40 cm

Participants evaluated the screen visibility at a distance of 30–40 cm, representing the typical distance between the screen and the operator during routine practice. Specifically, they were asked to give a score using a visual analog scale (VAS) (0–100 mm) to evaluate the visibility of the larynx displayed on the screen. In this study, a VAS of 0 indicated no visibility of the larynx, while a VAS of 100 represented perfect visibility of the larynx.

#### ***Preliminary experiment (***Fig. 2***)***

**Fig. 2:**
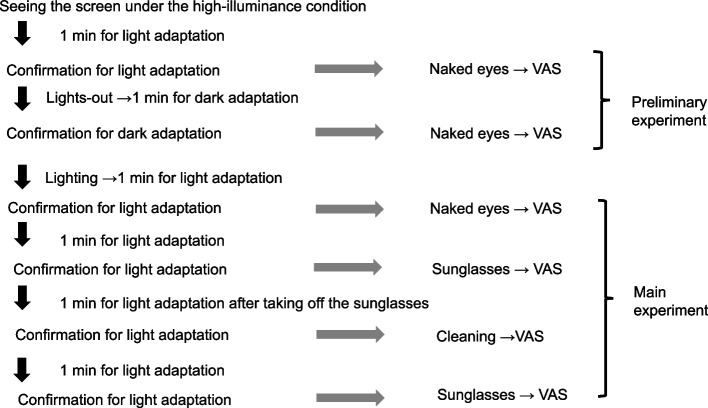
Experimental protocol. The experimental protocol consisted of two parts. The first part was a preliminary study to confirm whether the screen visibility on the video laryngoscope was impaired under a high-illuminance environment. Participants evaluated the visibility of the screen on the video laryngoscope under both high and usual luminance intensities. The second part was the main experiment to investigate whether each intervention could improve screen visibility on the video laryngoscope under a high-illuminance environment

Screen visibility on the video laryngoscope was assessed to determine whether it was impaired under high-illuminance conditions. During the evaluation, participants were instructed to view the screen while avoiding direct reflections from the floodlights. The position where each participant experienced the worst visibility was identified, and they were asked to maintain this position for all subsequent evaluations. However, it was confirmed that this worst viewing position remained stable even when the viewing angle varied slightly. This was crucial because later evaluations involved wearing and removing sunglasses.

To ensure consistency, adjustments were made by changing the monitor screen’s angle while keeping the typical distance between the screen and the operator. The VAS was assessed by having participants indicate a point on the VAS sheet as directed by the examiner, ensuring their head position remained stable. After this evaluation, the floodlights were turned off. Visibility was then reassessed under normal lighting conditions 1 min later. To confirm dark adaptation, participants were asked to read large newspaper letters placed on the monitor screen. This step was conducted to reconfirm that high-illuminance conditions impaired screen visibility on the video laryngoscope.

In a real outdoor setting on a sunny day, the overall brightness makes it difficult to see the video laryngoscope screen, regardless of the viewing angle, even if sunlight is not directly reflected. However, in the artificial setup simulating outdoor brightness, the bright area created by the floodlights was limited. As a result, visibility worsened only within a specific range of viewing angles. Therefore, the worst viewing position within the typical distance between the screen and the operator during routine use was selected.

#### ***Main experiment (***Fig. 2***)***

In a fixed order sequence comprising viewing with the naked eye, wearing sunglasses, cleaning the screen without wearing sunglasses, and cleaning the screen while wearing sunglasses, the main experiment to evaluate visibility with each intervention was conducted. Between each intervention, confirmation of light adaptation was performed using the naked eye as mentioned above because the effects of light adaptation might be compromised, especially after any intervention, including wearing sunglasses. Blinding each intervention was difficult, primarily due to the observable changes in screen condition after cleaning. Consequently, the order of interventions was not randomized. Initially, visibility was evaluated with the naked eye. In cases where participants wore glasses, the status of wearing glasses was considered as “naked eyes.” Subsequently, visibility was assessed with wearing sunglasses. A pair of polarized sunglasses (B0C6QYW331, OSCE Japan, Miyazaki, Japan), with no distinctive features and readily available, was used as the sunglass intervention. The functionality of the polarized lenses was confirmed using the included polarization test sheet, which contains images or text that become visible only when viewed through polarized lenses. In cases where participants wore glasses, they were asked to wear the sunglasses over their glasses. Following this evaluation, participants were instructed to remove the sunglasses. The monitor screen was then cleaned with a sheet of disinfecting wipes (Mikrozid universal wipes premium, Maruishi Pharmaceutical, Co., Ltd., Japan), and any remaining cleaning agent was wiped away with a sheet of dry tissue cloth (KimWipes, Kimberly-Clark, Irvine, USA). Under this condition, visibility was reassessed with the naked eye. Finally, visibility was reevaluated while wearing sunglasses.

### Sample size calculation

In this study, the VAS scores were treated as normally distributed continuous variables for the purpose of sample size calculation. Consequently, A paired *t*-test followed by Bonferroni’s correction was deemed appropriate to determine the required sample size. For the calculation, a difference of 20 mm with a common standard deviation of 20 mm was assumed for each comparison between interventions. The power analysis indicated that 22 volunteers would be required to achieve a statistical power of 0.95 with a type I error probability of 0.0083, where a type I error probability of 0.05 was adjusted for six comparisons using a two-tailed test. Ultimately, 28 volunteers were recruited to account for potential variations in estimation.

### Statistical analysis

The participant characteristics of the participants, including age, sex, years of clinical experience, and the use of corrective eyewear (including contact lenses), were summarized using descriptive statistics. All data were presented using the mean and standard deviation (SD) for normally distributed data, and the median and interquartile range (IQR) for non-normally distributed data, or as the number of participants. The VAS scores for visibility were presented as box plots, with scatter plots of individual values displayed alongside and assessed for their normality using Kolmogorov–Smirnov test. When a normal distribution was confirmed, to perform simple comparisons between and among the interventions, the paired *t*-test or the repeated measurement analysis of variance is followed by the paired *t*-test with the Bonferroni’s correction. When the null hypothesis for a normal distribution was rejected, the Wilcoxon signed-rank test or the Friedman test followed by the Wilcoxon signed-rank test with the Bonferroni’s correction. Analyses were computed using EZR (Saitama Medical Center, Jichi Medical University, Saitama, Japan). [6] EZR is a user-friendly interface for R (the R Foundation for Statistical Computing, Vienna, Austria). For all analyses, a significance level of *P* < 0.05 was considered as the threshold for statistical significance.

## Results

In this study, 28 anesthesiologists initially participated. However, four individuals who used sunglasses in their daily life were excluded. The mean age of the participants was 33 (8) years. The number of male and female participants was 11 and 13, respectively. The median clinical experience was 4 (1.8–8.0) years.

**Fig. 3:**
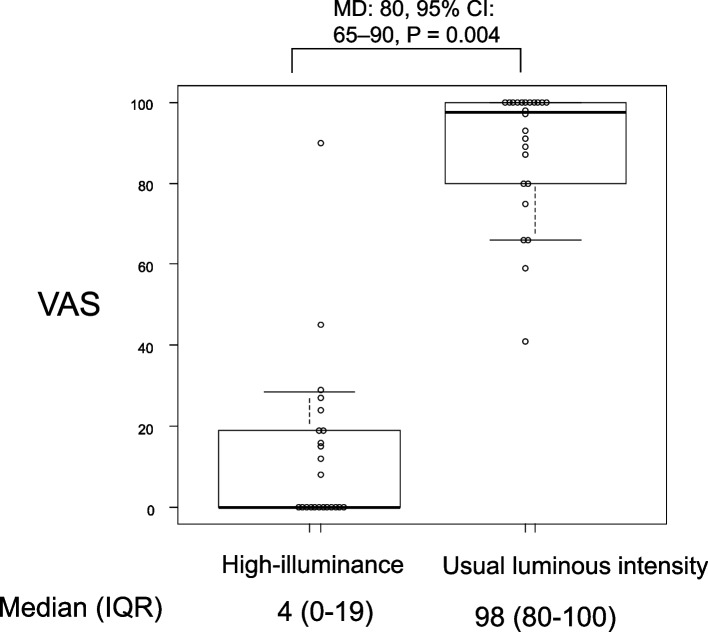
VAS scores for the visibility of the screen on the video laryngoscope under high-luminance intensity and usual luminance intensity. The box plot represents the median, quartiles, and the 10th and 90th percentiles, with a scatter plot of individual values displayed next to the box plot. VAS, visual analog scale; IQR, interquartile range; MD, median difference; 95% CI, 95% confidence interval

**Fig. 4:**
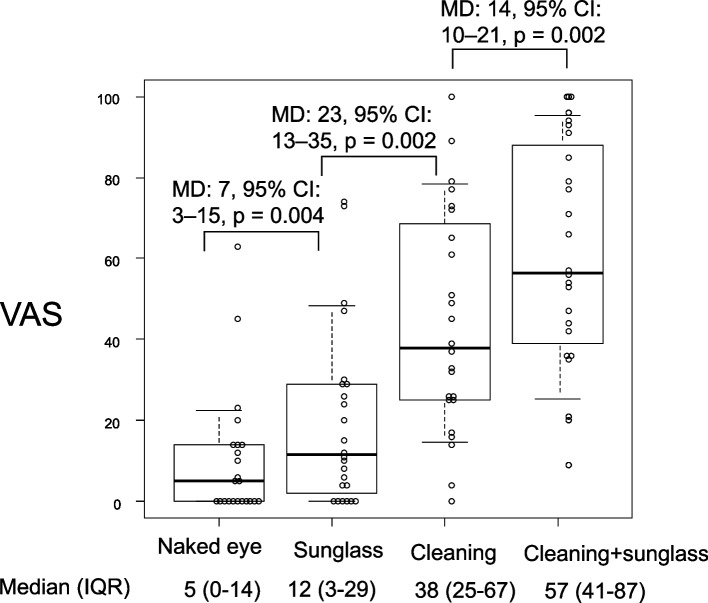
VAS scores for the visibility of the screen on the video laryngoscope under high-luminance intensity, including conditions with and without interventions. The box plot represents the median, quartiles, and the 10th and 90th percentiles, with a scatter plot of individual values displayed next to the box plot. All combination comparisons were significantly different; however, some *p*-values were omitted to avoid an overly busy figure. VAS, visual analog scale; IQR, interquartile range; MD, median difference; 95% CI, 95% confidence interval

The VAS scores for visibility were presented using boxplots and scatter plots (Figs. 3 and 4). Normal distribution was maintained only for the VAS scores evaluated under the conditions of cleaning the screen without wearing sunglasses and cleaning the screen while wearing sunglasses. Therefore, nonparametric analysis was used for comparing each intervention. In the preliminary experiment, the Wilcoxon signed-rank test was applied. In the main experiment, the Friedman test followed by the Wilcoxon signed-rank test with Bonferroni correction was applied. It was confirmed that the high-illuminance environment considerably impaired the visibility on the monitor screen, with a median VAS score of 4 compared to 98 (median difference (MD): 80, 95% confidence interval (CI): 65–90, *P* = 0.00002).

Regarding interventions to improve poor visibility, polarized sunglasses significantly enhanced visibility, with a median VAS score of 12 compared to 5 (*MD*: 7, 95% *CI*: 3–15, *P* = 0.004). Moreover, cleaning the monitor screen significantly improved visibility more than wearing sunglasses alone, with a median VAS score of 38 compared to 12 (*MD*: 23, 95% *CI*: 13–35, *P* = 0.002). Additionally, wearing sunglasses after cleaning the monitor screen provided even better visibility compared to only cleaning the screen, with a median VAS score of 57 compared to 38 (*MD*: 14, 95% *CI*: 10–21, *P* = 0.002).

## Discussion

From the results, it was confirmed that high-illuminance environment simulating outdoor conditions significantly impair visibility of video laryngoscope. Polarize sunglasses were effective to improve the visibility of video laryngoscope; however, a noteworthy fact was that cleaning the monitor screen was more effective to improve visibility than wearing sunglasses alone. This might be because the glare generated by high-illuminance conditions could be exacerbated by screen smudges to a degree that even polarized sunglasses cannot mitigate effectively. This phenomenon was likely attributable to the common experience that glare is exacerbated by screen smudges. In this condition, wearing polarized sunglasses further improved the visibility of video laryngoscope. It is reasonable to assume that polarized sunglasses could alleviate the glare caused by reflection or scattering on the display, which persisted even after cleaning in high-illumination conditions. [7]

Someone might want to know the effects of non-polarized sunglasses on the visibility of a display monitor. Non-polarized sunglasses and polarized sunglasses both mitigate outdoor light effectively. However, the main difference lies in their ability to filter polarized light. Most light sources emit unpolarized light, but light in nature can become polarized through reflection, absorption, and scattering. [8] Polarized sunglasses block much of the light reflected by surfaces, which significantly reduces glare in bright sunlight. [7] Therefore, non-polarized sunglasses would not be as effective as polarized sunglasses in enhancing the visibility of a display monitor under bright conditions. However, our study used glasses that both have tinting to reduce all transmitted light and are polarized. Therefore, it is undetermined which factor was more important in reducing the amount of light in sunny environments: polarization or tinting or both. To address this question, we need to assess non-polarized sunglasses and clear polarized lenses without tinting.

When using polarized sunglasses to view a liquid crystal display (LCD) monitor, special attention must be paid to the anti-glare function equipped on the monitor. Since the anti-glare function polarizes light, it may actually make the display harder to see. [9] Therefore, it is advisable to check beforehand if the video laryngoscope is equipped with an anti-glare function. In such cases, non-polarized sunglasses might be more suitable; however, cleaning the screen should be effective regardless of polarization.

There are several limitations to the current study. First, the interventions used in this study were not blinded to the participants. The interventions (wearing sunglasses and cleaning the screen) were obviously recognized by the participants as “an attempt to make the screen easier to see.” Therefore, it was difficult to blind this study. Although it was possible to mimic actual cleaning, it is believed to be challenging as the change in the display status following cleaning is easily noticeable. Second, the order of interventions was not randomized in this study. Therefore, the memory effects related to visualization of the larynx might have influenced the visibility in later interventions. However, the increase in VAS score with each intervention was so significant that we could not solely attribute the increase to the memory effect. In addition, the sequence from cleaning the screen to wearing sunglasses on a soiled screen proved practically difficult (requiring re-soiling the screen with artificial sweat and drying it). Therefore, randomization was abandoned. Additionally, the degree of dirtiness was considered to affect visibility, and reproducing the exact degree of dirtiness proved impractical. While randomization and blinding could have been implemented more effectively if multiple sets of experimental devices had been used instead of conducting sequential experiments with only one set, the limited number of available experimental devices prevented this. It is important to acknowledge that biases related to the interventions in this study were not fully mitigated. However, the significant improvement in visibility with each intervention was such that it could not be solely attributed to these biases, as mentioned earlier. Despite considering potential biases, the notable improvement in visibility suggests that these biases alone cannot fully explain the observed outcomes. Finally, it is uncertain whether the study environment fully replicated daylight conditions. However, in terms of brightness, the study conditions at least simulated daylight on a clear day. Therefore, we believe that the results demonstrate high reliability.

In conclusion, a high-illumination environment impaired the visibility of the video laryngoscope. Polarized sunglasses were effective in improving visibility; however, cleaning the monitor screen was found to be more effective. Wearing polarized sunglasses after cleaning the screen further enhanced visibility. Based on these findings, it is concluded that when using a video laryngoscope outdoors in sunny conditions, if visibility becomes impaired, the first step should be to clean the screen. Wearing sunglasses, if possible, can also be effective in improving visibility.

## Data Availability

The datasets used and analyzed during the current study are available from the corresponding author on reasonable request.

## Abbreviations

VAS: Visual analog scale
SD: Standard deviation
IQR: Interquartile range
LCD: Liquid crystal display
MD: Median difference
95% CI: Confidence interval
